# *Leucaena* interspecific hybrid ‘KX4-Hawaii’ as a source of agricultural biomass in a water-scarce small island developing state

**DOI:** 10.7717/peerj.18201

**Published:** 2024-09-26

**Authors:** Jabarry R. Belgrave, Angela T. Alleyne, Jeff S. Chandler, Francis B. Lopez

**Affiliations:** 1 The Department of Biological and Chemical Sciences, The University of the West Indies—Cave Hill Campus, Bridgetown, St. Michael, Barbados; 2 Formerly of The Department of Biological and Chemical Sciences, The University of the West Indies—Cave Hill Campus, Bridgetown, St. Michael, Barbados

**Keywords:** Agroforestry, Biomass, Leucaena, Hedgerow, KX4-Hawaii, Barbados

## Abstract

**Background:**

*Leucaena leucocephala* is a useful multipurpose tree species for agroforestry systems, but traditional seeded cultivars often become weedy and invasive. A seedless hybrid cultivar, ‘KX4-Hawaii’, offers a potential solution to this problem. However, relevant agronomic information and information on the performance of ‘KX4-Hawaii’ under varying growth conditions is required. The goal of this research was to evaluate ‘KX4-Hawaii’ as a source of agricultural biomass in Barbados, a small island developing state with limited arable land.

**Methods:**

‘KX4-Hawaii’ air layers were imported into Barbados to create stock trees. Air layering was used to create propagation material and a field study was established with a ‘KX4-Hawaii’ hedgerow planted as a field border. Three plant spacings (50, 75, and 100 cm) were evaluated and data on the growth and biomass yields of the trees were collected at 4-month intervals. Precipitation data were used to investigate climatic effects on ‘KX4-Hawaii’ productivity.

**Results:**

‘KX4-Hawaii’ was successfully propagated *via* air layers and could be planted directly in the field with irrigation. All recorded growth and biomass yields were correlated with precipitation. However, the woody (lignified stems and branches) biomass was more responsive to precipitation than the green (leaves and green tender stems) biomass and made up a large fraction of the total biomass produced. ‘KX4-Hawaii’ was productive even under drought conditions and biomass yields per meter of hedgerow increased with closer spacings. Of the tested spacing treatments, 75 cm was optimum for a 4-month pruning interval under the conditions seen in Barbados as it produced similar yields to the 50 cm spacing treatment but would require less propagation material.

## Introduction

**Figure 1:**
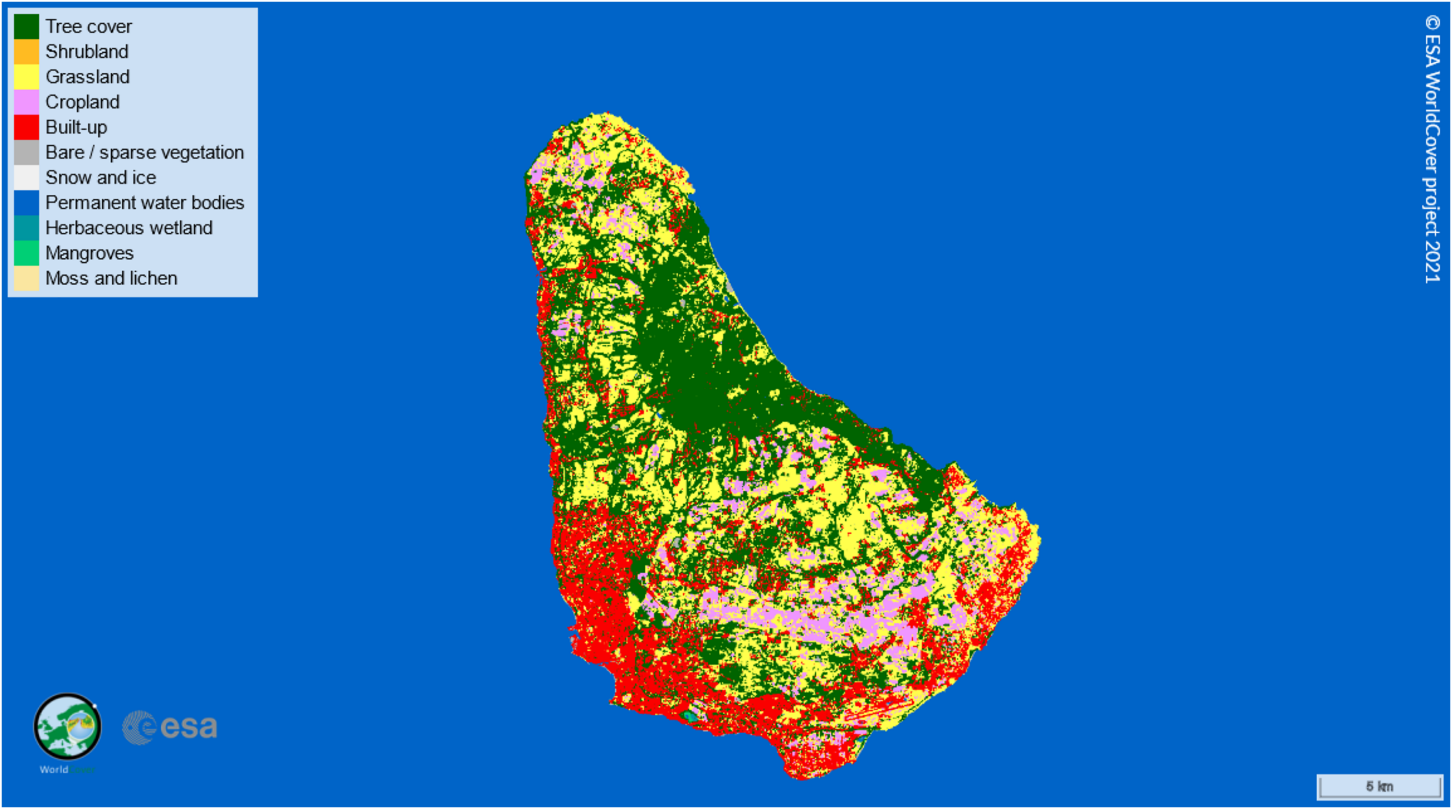
Map of Barbados showing land coverage. Arable crop land is indicated in pink. Map © 2024 ESA WorldCover Project at https://viewer.esa-worldcover.org/worldcover/.

Barbados is a small island developing state located in the Western Atlantic. It has a high food import bill (Madden, 2023) and worldwide ranks in the top 20 most water-scarce countries (FAO, 2015). The island is only 430 km^2^, and arable land coverage is heavily fragmented and has decreased over time, such that there were only 7,000 hectares in 2021 (The World Bank, 2024), as can be seen in Fig. 1 (Zanaga et al., 2022). Other Caribbean islands have similar conditions, leading to calls for sustainable and climate-smart agriculture in the region so that the limited resources available are used efficiently to improve food security (Central Bank of Barbados, 2023).

Several tropical woody tree species have multiple uses in agroforestry, including *Leucaena*, *Gliricidia*, *Inga*, and S*esbania* ssp., which can be found in Barbados (Carrington, 2007). They have been described as Multipurpose Tree Species (MPTs) for their use as bioenergy sources, food, and fodder, among other uses (Kang, Atta-krah & Reynolds, 1999; Verheij, 2007). Many MPTs are nitrogen fixers, and the fixed nitrogen may then be transferred to nearby crops *via* root exudation, leaf litter, and application of pruned biomass as mulch (Kang, Atta-krah & Reynolds, 1999; Youkhana & Idol, 2009). Nitrogen-fixing MPTs, such as Leucaena (*Leucaena leucocephala* (Lam.) de Wit), may be used in agroforestry to produce biomass for soil amelioration during crop cultivation, and their biomass may be pruned and added to the soil as a mulch or mixed into the soil as a green manure. These soil additions may lead to improvements in soil organic matter, water retention, mineral nutrients, and reductions in nutrient leaching (Kang, Atta-krah & Reynolds, 1999; Youkhana & Idol, 2008). Organically improved soils result in more sustainable and resilient agroecosystems (requiring fewer external chemical inputs), particularly due to climate change effects such as floods and droughts, which influence soil erosion, and water and nutrient availability (Kugedera, Mandumbu & Nyamadzawo, 2022; Lalljee, 2013).

When MPTs are grown in a different location to the crop, and the pruned biomass is carried to the crop to be applied, the process is described as a cut and carry or biomass transfer system (Kang, Atta-krah & Reynolds, 1999; Youkhana & Idol, 2017). Cut and carry systems require more human resources to transport the pruned biomass, although the MPTs can be grown on less arable land, leaving prime arable land for crop cultivation. Still, cut and carry systems eliminate competition between MPTs and crops unlike other agroforestry techniques such as alley cropping. Consequently, research has shown that cut and carry systems can increase crop productivity. A cut and carry system using Leucaena, Gliricidia (*Gliricidia sepium* (Jacq.) Walp) and Senna (*Senna siamea* (Lam.) Irwin et Barneby), resulted in higher yields than control or fertilizer treatments, but was uneconomical due to high labor costs and low land availability in Nigeria (Kormawa et al., 1999). Contrastingly, a cut and carry system in Southeast Asia using mountain immortelle (*Erythrina poeppigiana* (Walp.) Cook) to produce beans (*Phaseolus vulgaris* L.) and maize (*Zea mays* L.) resulted in greater yields and was more profitable than alley cropping (Prosea Foundation, 1997). A cut and carry system in Hawaii using mulch from Leucaena ‘KX2’ to produce coffee (*Coffea arabica* L.) also resulted in increased coffee growth and yield and soil carbon and nitrogen contents (Youkhana & Idol, 2016).

Higher green (non-lignified leaf and stem) biomass production is helpful for soil nutrient additions, as lower carbon-to-nitrogen (C:N) ratios result in faster biomass decomposition when biomass is applied to the soil as a mulch (Kang, Atta-krah & Reynolds, 1999; Youkhana & Idol, 2009). Low C:N ratios have also been linked to increased biomass quality and crop yields (Srivastava & Singh, 2013; Tian, Brussaard & Kang, 1995; Vargas-Tierras et al., 2021). However, woody biomass with higher C:N ratios can be used as a mulch that is more resistant to decomposition than green biomass and can reduce soil evaporative water loss for longer periods (Budelman, 1988; Youkhana & Idol, 2016). The configuration of a Leucaena hedgerow can be altered to change the dominant fractions in the pruned biomass to suit the needs of the grower if prior knowledge about its use and responses is available.

Research in Hawaii has found that more frequent pruning and wider spacings increased Leucaena green biomass yields (t/ha) (Guevarr, 1976). Contrastingly, later studies reported that wider plant spacings led to decreased green and woody Leucaena biomass yields (Chotchutima et al., 2013; Tuncay & Rüstü, 1989). This effect was also reported by Elfeel & Elmagboul (2016) who reported increased green and woody biomass yields (t/ha) overall with closer spacing, but decreased woody biomass content.

‘KX4 Hawaii’ is a triploid seedless Leucaena interspecific hybrid (*L. leucocephala* ssp. *glabrata* (Rose) S. Za´rate ‘K636’ and *L. esculenta* (Mocin˜o et Sesse ex DC) Benth.*L* ‘K838’) with high biomass production levels (Brewbaker, 2013). It is a giant Leucaena, like its *L. leucocephala* ssp. *glabrata* parent, and can grow to a height of 20 m, unlike common Leucaena (*L. leucocephala* subsp. *leucocephala*) which has a shrubby growth habit, higher reproductive output, and is more invasive (Bageel et al., 2020; Brewbaker, 2016). Giant Leucaena can be induced to keep a manageable shrub-like form by pruning multiple times per year. Leucaena is considered invasive in Barbados, and farmers are hesitant to grow it because after its introduction to Barbados it escaped and became widespread across the island with a high reproductive output (Carrington, 2007; Proverbs, 1984). However, these Leucaena are self-pollinated seeded types with a maximum of 45 pods per flower head, each containing 8–18 seeds, while flowering and fruiting year-round (Orwa et al., 2009). Seedless cultivars offer a possible solution to this concern. They are less likely to escape and become invasive, but little information is available on their productivity in an agronomic setting.

The ‘KX4-Hawaii’ hybrid was released in 2012 and it is a completely seedless and high-yielding Leucaena hybrid (Brewbaker, 2013). ‘KX4-Hawaii’ may also have potential to supplant the invasive local Leucaena in Barbados for cut stakes, fish traps, biochar, animal feed, land stabilization, and agronomic purposes (Carrington, 2007; Proverbs, 1984), without the risk of it becoming feral.

This study sought to evaluate the seedless cultivar of Leucaena (cv. ‘KX4-Hawaii’), and the effect of intra-row plant spacing on biomass yields when planted as a hedgerow on a field border in Barbados. Both green and woody biomass yields were considered, as green biomass has applications for animal feeds and soil improvements, while woody biomass has applications for wood production, biochar, and biomass to energy, and Leucaena has been used in these roles locally (Carrington, 2007; Proverbs, 1984). As Barbados is a small water scarce island, the performance of this cultivar under these conditions needs to be established.

## Materials and Methods

### Establishment of Leucaena ‘KX4-Hawaii’

Forty, 30-cm-long rooted air layers of Leucaena cultivar ‘KX4-Hawaii’ were imported into Barbados from the University of Hawaii in 2015. These were acclimated in the Biology Gardens at the Cave Hill Campus in plastic nursery pots (33 cm diameter, 25 cm high) filled with a 1:1 ratio of loamy topsoil and construction sand with daily irrigation. The surviving air layers (25) were planted at the Barbados Agriculture and Manufacturing Company’s (BAMC) research station at Groves, Saint George, Barbados to establish stock trees. These were spaced 2 m apart and 15 g of triple super phosphate fertilizer was placed in each planting hole.

After the stock trees had been established for 2 years, 240 air layers were created by removing 3 cm wide strips of bark from branches 4 to 6 cm in diameter, and cheese cloth containing 125 cm^3^ of moist Sphagnum peat moss was applied, before wrapping the wounded portion with plastic wrap. Up to three air layers were initiated per branch and the branches were cut after 5 weeks to produce 40 cm long air layers. These were acclimated as was done with the imported ‘KX4-Hawaii’ air layers and 183 air layers (76.25%) survived. Further air layers were created (cut after 8 weeks) and these were planted directly in the field without acclimatization in pots to test the feasibility of skipping the nursery stage of propagation. Of the 39 air layers directly planted, 33 survived (84.6%).

### Experimental field plot and planting

A field experiment was established at the Barbados Ministry of Agriculture’s Graeme Hall complex, in the parish of Christ Church, (13.074379°N 59.573093°W), in 2018. This site is located 11 m above sea level in Barbados’ black soil association, characterized by fine textured clays (Ahmad, 2011). A randomized block design (with five blocks arranged linearly along a single hedgerow on one continuous ridge) was used to evaluate the plant spacing (50, 75 and 100 cm) between ‘KX4-Hawaii’ trees planted as a field border hedgerow. The ridge was 1.2 m wide. There were three plots per block (the position of each plot within each block was randomized), each plot was 9 m long, and plots were spaced 2 m apart for a total block length of 33 m. Each 50, 75, and 100 cm spacing plot contained 18, 12 and 9 plants, respectively, for a total of 39 plots per block. Due to seasonal variations such as the dry season in Barbados between December and May (Mohan, Clarke & Chadee, 2020), a 4-month pruning interval was chosen so that adequate yields could still be acquired during dry periods when Leucaena biomass production drops (Proverbs, 1984). After each pruning a 0.15% Roundup Ultra herbicide solution (a.i. glyphosate, Bayer AG, Germany) was spot sprayed to control weeds based on previously reported excellent weed control and minimum Leucaena mortality using this technique in Barbados (Proverbs, 1984).

Experimental blocks 1–4 were planted using the 183 acclimated air layers. As more land was available at the study site than initially discussed with the Agriculture Ministry, a fifth block (originally unplanned) was planted using the 39 air layers directly planted in the field to utilize the additional land. Supplying was done 2 weeks after planting. The trees were established for 7 (blocks 1–4) or 9 months (block 5), with daily drip irrigation for the first month. Then they were pruned to 30 cm to standardize the plants to the same height in September 2018 for blocks 1 to 4 and in January 2019 for block 5.

### Biomass data collection

There were four prunings (spanning 16 months) until the experiment was prematurely concluded due to COVID-19 lockdowns in Barbados in 2020 (Table 1). Block 5 was established later than the other blocks and data were not collected from this block until May 2019 (13 months after planting). Apart from the first interval (ending January 2019) the amount of precipitation received was low.

**Table 1:** Data collection dates, and the duration and the amount of precipitation received, for each hedgerow pruning interval. Measurements activities refer to recording plant height, canopy width and canopy light interception. Sampling activities refer to biomass yield sampling. The “Previous Cut” date refers to the date after all sampling and measurements were concluded when all hedgerow trees were cut to a 30 cm height. The precipitation data are from the Charnocks (Grantley Adams International Airport) weather station, which was the station closest to the study site, and are the cumulative rainfall received between the “Previous Cut” date and the measurements and sampling date(s).

Pruning	Blocks	Previous cut	Activity	Date	Duration (days)	Precipitation per duration (mm)
1 (Jan 2019)	All	2018.09.08	Measurements & sampling	2019.01.06	120	576.0
2 (May 2019)	All	2019.01.07	Measurements	2019.05.02	115	150.5
	All	2019.01.07	Sampling	2019.05.04	117	150.5
3 (Sep 2019)	1–2	2019.05.14	Measurements	2019.09.05	116	277.1
	3–5	2019.05.12	Measurements	2019.09.05	114	277.4
	1–2	2019.05.14	Sampling	2019.09.07	118	296.9
	3–5	2019.05.12	Sampling	2019.09.07	116	297.2
4 (Jan 2020)	1–2	2019.09.18	Measurements	2020.01.10	114	266.3
	3–5	2019.09.09	Measurements	2020.01.10	123	284.7
	1–2	2019.09.18	Sampling	2020.01.09	113	265.1
	3–5	2019.09.09	Sampling	2020.01.09	122	283.5

The plant height and canopy width of two plants in the center of each plot were recorded and averaged, and biomass samples were taken from a single plant in the center of each plot (resources to process biomass samples from two plants per plot were not available). Data were collected from plants in the center of each plot to reduce any edge effects on the results. Plant height and canopy width (perpendicular across hedgerow) were measured with a meter ruler. Light interception was measured using a LI-190R point quantum sensor and one meter LI 191R line quantum sensor (LI COR, Inc., Lincoln, NE, USA) between 10:00 and 12:00 am. The line sensor was centered on the ground below the plant in each plot to be used for biomass sampling and measurements were only taken if the overhead light was at least 1,000 μmol s^−1^ m^−2^.

Plots were pruned to 30 cm high and biomass samples were taken approximately every 4 months. All pruned biomass from a single plant (due to limited resources sampling two plants per block would not be possible) from the center of each plot was used to determine the green biomass (leaves and green tender stems ≤ 1 cm in diameter) and woody biomass (more lignified stems and branches > 1 cm in diameter) yield per plant. The fresh weight of each sample was recorded, subsamples were weighed and oven-dried at 105 °C until constant weight, and the dry weight of the subsamples were used to determine the dry mass of the samples.

### Data analysis

Block 2 was not analyzed due to very shallow soil depths in that area of the hedgerow that resulted in plots 4 and 5, and half of plot 6, performing extremely poorly. The ‘jamovi’ 2.5.3 software with the GAMLj: General analyses for linear models 3.2.7 module was used for mixed model, correlation, and regression analyses (Gallucci, 2019). A log10 transformation/back transformation was used during mixed model analysis if the residuals were not normally distributed (canopy light interception and the biomass yield variables), and polynomial contrasts were applied during mixed modelling for trend analysis. The Tukey honest significant difference *post hoc* test was applied to significant (*p* ≤ 0.05) mixed model results for pairwise mean comparisons. The strength of correlations was defined based on Swinscow (1997).

## Results

### ‘KX4-Hawaii’ growth

During 2019, seasonal variations were observed for some growth parameters Plant height, and canopy light interception were at their highest on the January 2019, and canopy width was highest in September 2019, though not significantly different to January 2019 (Table 2). Plant height, canopy width and canopy light interception were at their lowest in May 2019 during the dry season. Although differences between the mean plant height achieved were significant throughout the sampling period, there were no significant differences in canopy width and light interception between the January 2019 and September 2019 sampling dates. There was also no significant difference in canopy light interception between the May 2019 and January 2020 sampling dates, although significantly wider canopies were recorded in January 2020 than in May 2019.

**Table 2:** Leucaena plant height, canopy width, and canopy light interception at each pruning date ± standard error from linear mixed model analyses.

Time (sampling date)	Plant height (cm)	Canopy width (cm)	Canopy light interception (%)
January 2019	284 ± 6.88d	273 ± 9.34c	89.13 ± 2.59b
May 2019	130 ± 6.09a	152 ± 8.56a	69.18 ± 2.01a
September 2019	259 ± 6.09c	292 ± 8.56c	81.28 ± 2.53b
January 2020	203 ± 6.09b	213 ± 8.56b	67.61 ± 2.10a

**Note:**

Plant height F = 167.32, d.f. = 3, *n* = 45, *p* < 0.001. Canopy width F = 114.70, d.f. = 3, *p* < 0.001. Canopy light interception F = 17.61, d.f. = 3, *p* < 0.001. Means in the same column with a common attached letter are not statistically different based on the Tukey HSD *post hoc* test (*p* ≤ 0.05).

The 75 cm spacing treatment consistently resulted in greater plant heights and canopy light interception (Table 3). The 75 cm spacing treatment was significantly different to the 100 cm spacing treatment in these variables, although it was not significantly different to the 50 cm spacing treatment. Similarly, wider canopy widths were observed with a 75 cm spacing interval. At the 50, 75, and 100 cm spacing intervals the mean canopy widths were 222 ± 10.1, 255 ± 10.1, and 220 ± 10.1 cm, respectively, but the linear mixed model results were not significant (F = 4.77, d.f. = 2, *p* = 0.060). There was a quadratic response of plant height (*p* = 0.013), canopy width (*p* = 0.023), and light interception (*p* < 0.001) as plant spacing increased. There was also a linear response of canopy light interception to plant spacing (*p* < 0.001).

**Table 3:** Leucaena plant height and canopy light interception for each spacing treatment ± standard error from linear mixed model analyses.

Spacing (cm)	Plant height (cm)	Canopy light interception (%)
50	211 ± 7.48ab	81.28 ± 1.91b
75	238 ± 7.48b	85.11 ± 2.00b
100	208 ± 7.48a	69.18 ± 1.63a

**Note:**

Plant height F = 4.86, d.f. = 2, *n* = 45, *p* = 0.039. Canopy light interception F = 17.92, d.f. = 2, *p* < 0.001. Means in column with a common attached letter are not statistically different based on the Tukey HSD *post hoc* test (*p* ≤ 0.05).

### ‘KX4-Hawaii’ biomass yields

The yield per plant at each sampling date for each spacing treatment can be found in Table 4. These yields were equivalent to 27.94 Mg/ha/year green biomass and 47.19 Mg/ha/year woody biomass for the 50 cm spacing treatment, 23.38 Mg/ha/year green biomass and 44.19 Mg/ha/year woody biomass for the 75 cm spacing treatment, and 13. 88 Mg/ha/year green biomass and 24.63 Mg/ha/year woody biomass for the 100 cm spacing treatment.

**Table 4:** Leucaena biomass yields per plant (dry weight) at each pruning date ± standard error.

Time (sampling date)	Spacing (cm)	Green biomass yield/plant (kg)	Woody biomass yield/plant (kg)
January 2019	50	0.70 ± 0.24	1.58 ± 0.24
	75	0.77 ± 0.24	1.83 ± 0.24
	100	0.56 ± 0.24	1.50 ± 0.24
May 2019	50	0.56 ± 0.22	0.65 ± 0.22
	75	0.61 ± 0.22	0.80 ± 0.22
	100	0.53 ± 0.22	0.62 ± 0.22
September 2019	50	0.62 ± 0.22	0.88 ± 0.22
	75	0.84 ± 0.22	1.30 ± 0.22
	100	0.74 ± 0.22	1.10 ± 0.22
January 2020	50	0.36 ± 0.22	0.59 ± 0.22
	75	0.59 ± 0.22	1.33 ± 0.22
	100	0.39 ± 0.22	0.72 ± 0.22

**Note:**

*n* = 135.

Green biomass comprised a lower fraction of the total biomass produced by ‘KX4-Hawaii’ (Table 5) and its yields were significantly different to the woody biomass yields at each sampling date apart from May 2019. Yet, overall biomass yields recorded in May 2019 did not differ significantly to those recorded in January 2020. However, the woody biomass yields were significantly different between January and September 2019.

**Table 5:** Leucaena biomass yields (dry weight) for each biomass component at each pruning date ± standard error from linear mixed model analyses.

Time (sampling date)	Biomass type	Yield/plant (kg)	Yield/meter of hedgerow (kg)
January 2019	Green	0.66 ± 0.08bc	0.91 ± 0.11bc
	Woody	1.56 ± 0.18fg	2.17 ± 0.25fg
	Total	2.22 ± 0.26g	3.08 ± 0.36g
May 2019	Green	0.56 ± 0.06ab	0.77 ± 0.08ab
	Woody	0.68 ± 0.07bc	0.94 ± 0.10bc
	Total	1.23 ± 0.13ef	1.71 ± 0.19ef
September 2019	Green	0.69 ± 0.08bc	0.96 ± 0.10bc
	Woody	1.04 ± 0.11de	1.44 ± 0.16de
	Total	1.74 ± 0.19g	2.41 ± 0.26g
January 2020	Green	0.40 ± 0.04a	0.56 ± 0.06a
	Woody	0.78 ± 0.09cd	1.08 ± 0.12cd
	Total	1.19 ± 0.13ef	1.65 ± 0.18ef

**Note:**

F = 3.86, d.f. = 6, *n* = 135, *p* = 0.002. Means in the same column with a common attached letter are not statistically different based on the Tukey HSD *post hoc* test (*p* ≤ 0.05).

The 75 cm spacing treatment resulted in consistently higher yields per plant, although this was not significant. There were no significant effects of the spacing×date interaction on ‘KX4-Hawaii’ biomass yields and spacing only significantly affected the biomass yields per meter of hedgerow (F = 6.78, d.f. = 2, *p* = 0.016). The 50, 75 and 100 cm spacing treatments resulted in a mean total biomass production of 2.70 ± 0.43, 2.54 ± 0.40, and 1.43 ± 0.23 kg per meter of hedgerow respectively. Biomass yields per meter of hedgerow decreased with higher spacings and polynomial contrasts indicated a significant linear response of biomass yield per meter to spacing (*p* = 0.009). Notably, the 50 and 75 cm spacing treatments did not significantly differ in biomass yields per meter (*p* = 0.021), but both resulted in significantly different biomass yields per meter to the 100 cm treatment.

There was a quadratic response (contrast) of the green/woody biomass ratio to plant spacing (*p* = 0.048) but the mixed model results were not significant (F = 2.69, d.f. = 2, *p* = 0.085). However, there was a significant effect of date on the green/woody biomass ratio (F = 64.13, d.f. = 3, *p* < 0.001) and the ratio was significantly different between all sampling dates. The green/woody biomass ratio was 0.41 ± 0.03 in January 2019, increasing to 0.83 ± 0.03 in May 2019, and declining to 0.67 ± 0.03 in September 2019 and to 0.524 ± 0.03 in January 2020.

### Associations between spacing, precipitation and growth parameters with ‘KX4-Hawaii’ growth and yield

The growth parameters measured were significantly correlated with biomass yield (Table 6). There were moderate positive correlations between plant height, canopy width, and light interception with green biomass yield per plant. Correlations between these variables and the woody biomass yield per plant were strong and positive, apart from that with canopy light interception which was moderate in strength (rho = 0.499, d.f. = 2, *p* < 0.001). All correlations between the recorded growth parameters and the green:woody biomass ratio were negative and the strength was weak for canopy light interception (rho = −0.346, d.f. = 42, *p* = 0.022), moderate for canopy width (rho = −0.455, d.f. = 43, *p* = 0.002), and strong for plant height (rho = −0.645, d.f. = 43, *p* < 0.001).

**Table 6:** Spearman’s rank correlations between the recorded growth and yield variables.

Parameter	Green biomass yield per plant (kg)	Woody biomass yield per plant (kg)	Green: woody biomass ratio (kg/kg)
Plant height (cm)	rho = 0.433	rho = 0.693	rho = −0.645
	d.f. = 43	d.f. = 43	d.f. = 43
	*p* = 0.003	*p* < 0.001	*p* < 0.001
Canopy width (cm)	rho = 0.517	rho = 0.662	rho = −0.455
	d.f. = 43	d.f. = 43	d.f. = 43
	*p* < 0.001	*p* < 0.001	*p* = 0.002
Canopy light interception (%)	rho = 0.411	rho = 0.499	rho = −0.346
	d.f. = 42	d.f. = 42	d.f. = 42
	*p* = 0.005	*p* < 0.001	*p* = 0.022

**Note:**

*n* = 45 (*n* = 44 for correlations with canopy light interception due to one missing data point).

When the data for each spacing treatment were pooled, all recorded growth and biomass yield variables were significantly correlated with the cumulative precipitation received during each cutting interval (Table 7). These correlations were all positive, apart from the strong negative correlation with the green:woody biomass ratio (rho = −0.686, d.f. = 43, *p* < 0,001). Notably, the correlation with plant height (rho = 0.757, d.f. = 43, p < 0.001) was strong as compared to the moderate correlations with canopy width (rho = 0.581, d.f. = 42, *p* < 0.001) and light interception (rho = 0.434, d.f. = 42, *p* = 0.003). The correlation with green biomass yields was weak (rho = 0.375, d.f. 43, *p* = 0.011) compared to the strong correlation with woody biomass yields (rho = 0.641, d.f. = 43, *p* < 0.001). There were no significant correlations between any recorded variable and the length of the interval between data collection dates apart from the moderate negative correlation with the green:woody biomass ratio (rho = −0.516, *N* = 43, *p* < 0.001). When correlation analyses were conducted using data from each spacing treatment individually, the canopy light interception of the 75 cm spacing treatment was not significantly correlated with precipitation (rho = 0.415, d.f. = 13, *p* = 0.124), and the green biomass yield per plant of the 50, 75 and 100 cm treatments were not significantly correlated with precipitation (rho = 0.300, d.f. = 13, *p* = 0.277; rho = 0.411, d.f. = 13, *p* = 0.128; and rho = 0.419, d.f. = 13, *p* = 0.120 respectively) (Table 7).

**Table 7:** Spearman’s rank correlations between precipitation and the recorded growth and yield variables.

Spacing treatment (cm)	Plant height (cm)	Canopy width (cm)	Canopy light interception (%)	Green biomass yield per plant (kg)	Woody biomass yield per plant (kg)	Green: woody biomass ratio (kg/kg)
All (Pooled)	rho = 0.757	rho = 0.581	rho = 0.434	rho = 0.375	rho = 0.641	rho = −0.686
	d.f. = 43	d.f. = 43	d.f. = 42	d.f. = 43	d.f. = 43	d.f. = 43
	*p* < 0.001	*p* < 0.001	*p* = 0.003	*p* = 0.011	*p* < 0.001	*p* < 0.001
50	rho = 0.711	rho = 0.623	rho = 0.568	rho = 0.300	rho = 0.520	rho = −0.732
	d.f. = 13	d.f. = 13	d.f. = 12	d.f. = 13	d.f. = 13	d.f. = 13
	*p* = 0.003	*p* = 0.013	*p* = 0.034	*p* = 0.277	*p* = 0.047	*p* = 0.002
75	rho = 0.775	rho = 0.548	rho = 0.415	rho = 0.411	rho = 0.715	rho = −0.594
	d.f. = 13	d.f. = 13	d.f. = 13	d.f. = 13	d.f. = 13	d.f. = 13
	*p* < 0.001	*p* < 0.034	*p* = 0.124	*p* = 0.128	*p* = 0.003	*p* = 0.020
100	rho = 0.813	rho = 0.638	rho = 0.638	rho = 0.419	rho = 0.796	rho = −0.769
	d.f. = 13	d.f. = 13	d.f. = 13	d.f. = 13	d.f. = 13	d.f. = 13
	*p* < 0.001	*p* = 0.011	*p* = 0.013	*p* = 0.120	*p* < 0.001	*p* < 0.001

**Note:**

*n* = 15.

Multiple linear regression modelling of spacing, spacing^2^, and the duration of each cutting interval, and the amount of precipitation received was not significant when fitted to the green biomass yield per plant (adjusted R^2^ = 0.100, d.f. = 4,40, *p* = 0.083) (Table 8). However, the regression model fitted to the woody biomass yield per plant was significant (adjusted R^2^ = 0.100, d.f. = 4,40, *p* = 0.083) and spacing (*p* = 0.004), spacing^2^ (*p* = 0.005), and precipitation (*p* < 0.001) were significant predictors of woody biomass yield. This model explained 47.9% of the variation in woody biomass yields. However, the duration of the interval between cuts (*p* = 0.508) was not a significant predictor of woody biomass yield, likely because the intervals only ranged from 113 to 123 days; a maximum difference of 10 days. There was a quadratic response of woody biomass yield per plant to plant spacing (spacing, B = 8.96e−2; spacing^2^, B = −5.87e−4), indicating that yield increased initially with spacing before declining. Woody biomass yields also increased with increasing precipitation (B = 2.23e−3).

**Table 8:** Multiple linear regression models of green and woody biomass yields per plant fitted to plant spacing, plant spacing squared, and the duration and the amount of precipitation received for each cutting interval.

Variable	Model fit	Predictor	Estimate (B)	S.E.	t	*p*
Green biomass yield per plant (kg)	R^2^ = 0.100	Intercept	0.82	1.644	0.50	0.620
	d.f. = 4,40	Spacing (cm)	3.60e−2	1.60e−2	2.23	0.031
	*p* = 0.083	Spacing (cm)^2^	−2.36e−4	1.07e−4	−2.20	0.033
		Precipitation (mm)	4.59e−4	2.36e−4	1.95	0.058
		Interval (Days)	−1.39e−2	1.30e−2	−1.04	0.303
Woody biomass yield per plant (kg)	R^2^ = 0.479	Intercept	−4.73	3.01	−1.57	0.124
	d.f. = 4,40	Spacing (cm)	8.96e−2	2.96e−2	3.03	0.004
	*p* < 0.001	Spacing (cm)^2^	−5.87e−4	1.96e−4	−2.99	0.005
		Precipitation (mm)	2.23e−3	4.31e−4	5.16	<0.001
		Interval (Days)	1.62e−2	2.42e−2	0.67	0.508

**Note:**

*n* = 45, R^2^ = adjusted R^2^ statistic.

## Discussion

### Propagation of Leucaena ‘KX4-Hawaii’

Air layering is a low-tech method of propagation with a higher than 90% success rate for ‘KX4-Hawaii’ propagation under ideal conditions (Idol, Youkhana & Santiago, 2019), so it was used to produce ‘KX4-Hawaii’ planting material in this study. However, the survival rates (76.25% and 84.6%) achieved were lower than the 90% success rate reported by Idol, Youkhana & Santiago (2019). It should be considered that the rooted air layers directly planted in the field, though irrigated initially, were propagated under rain-fed conditions and this may explain the lower success rates. Any differences between these findings and those of Idol, Youkhana & Santiago (2019) in Hawaii might be due to the use of larger diameter branches for air layering in this study. Idol, Youkhana & Santiago (2019) also mentioned that adequate soil moisture was needed for the best success rate in producing rooted air layers, and the stock trees used in this work were not irrigated. Furthermore, Hawaii is a volcanic island with volcanic soils, while Barbados’ soils are mainly of limestone origin (Ahmad, 2011). Generally, there is agronomic interest in modern seedless Leucaena cultivars, but breeders should consider more efficient multiplication methods for large scale distribution and planting.

### Effects of plant spacing on ‘KX4-Hawaii’ growth and yield

Overall, the 75 cm spacing resulted in the greatest ‘KX4-Hawaii’ growth and biomass production on aper-plant basis. Research has shown that Leucaena does not compensate for wider plant spacings by producing more lateral stems (Guevarr, 1976). Therefore, when space is no longer a limiting factor, further stem growth that would normally drive a bigger canopy and greater biomass does not occur. Leucaena biomass yield per plant (effects of experimental treatments at the individual plant level) and per unit area or length of hedgerow (yields under a production setting) are both helpful metrics to measure plant productivity in response to experimental treatments. Our results showed that there were no significant effects of plant spacing on the biomass yield per plant, but there were significant effects of plant spacing on the biomass yield per meter of hedgerow. Previous research has shown that Leucaena biomass yield per plant did not significantly increase with intra-row plant spacings above 25 cm (Chotchutima et al., 2013; Tuncay & Rüstü, 1989), and 50 cm was the smallest plant spacing used in this current study. As plant spacing did not significantly affect biomass yield per plant, a higher biomass yield per length of hedgerow with smaller plant spacings was due to greater plant densities.

The green:woody biomass ratio responded quadratically to plant spacing and the 75 cm spacing treatment resulted in the lowest green:woody biomass ratio (it had the largest proportion of woody biomass). This was unexpected, as literature sources suggest narrower plant spacings are superior for woody biomass production (Chotchutima et al., 2013; Elfeel & Elmagboul, 2016; Prasad et al., 2011). Another factor in the lower biomass yield per plant of the 100 cm plant spacing treatment might have been because it took longer for this treatment to form a solid canopy and shade out weeds (if it ever did within 4 months). This allowed more weeds to grow inside the hedgerow, which would lead to interspecific competition that would counteract the lower intraspecific competition due to a lower plant density. *Desmanthus virgatus* (L.) Willd and *Bothriochloa pertusa* (L.) A.Camus were common weeds in the ‘KX4-Hawaii’ hedgerow in this study.

The biomass yield per meter of hedgerow responded linearly to plant spacing, with an inverse relationship between plant spacing and biomass yields. The 75 cm spacing treatment can be recommended for ‘KX4-Hawaii’ under similar conditions in the present study as it did not differ significantly from the yields per meter of hedgerow of the 50 cm spacing interval but would require less planting material due to the smaller population produced. Our findings are similar to those by Elfeel & Elmagboul (2016), who also used a 4-month pruning interval across four biomass sampling dates. Biomass yields recorded from their first biomass sampling responded quadratically to plant spacing and linearly to plant spacing on the subsequent three sampling dates, with the lowest plant spacing (40 cm) having higher biomass yields. On its initial release, the ‘KX4-Hawaii’ cultivar was noted for its wood and biofuel production (Brewbaker, 2013). However, earlier studies with Leucaena by Guevarr (1976) reported that green biomass comprised the predominant fraction when compared to woody biomass yields. This differed from later studies by Elfeel & Elmagboul (2016) who reported the production of equal proportions of Leucaena green and woody biomass. In the current study, woody biomass comprised the predominant fraction of the ‘KX4-Hawaii’ biomass produced. Therefore, the high woody biomass production here makes ‘KX4-Hawii’ a suitable cultivar under these conditions as a biofuel source.

### Impacts of climate conditions during the study period

Leucaena can grow with 650 mm of annual precipitation (Orwa et al., 2009; Proverbs, 1984) but this amount is not optimal. Leucaena has been shown to respond to water availability (Maslekar, 1984; Noulèkoun et al., 2017) and thrives with over 1,200 mm of annual precipitation (Orwa et al., 2009). However, there was a drought during the study period in 2019 in Barbados (Alleyne, 2020; Smith, 2020), with the Charnocks weather station receiving only 58% (736.5 mm) of the climatological average precipitation. This was the lowest amount of precipitation received since 1942 (Alleyne, 2020). Barbados’ climatological average of 1,260 mm of annual precipitation (Layne, 2021) falls into a favorable precipitation regime for Leucaena growth. Therefore, there is potential for greater ‘KX4-Hawaii’ growth and biomass production than that seen in 2019 outside of drought conditions. Notably, the September 6th to December 31st period in 2018 (580.4 mm) was not dissimilar to the climatological average of 1991–2020 for the September to December period (605.8 mm) (Layne, 2021), and this period resulted in the highest woody and total biomass yields when the Leucaena trees were pruned in early January 2019. There were positive correlations between precipitation and all recorded ‘KX4-Hawaii’ growth and yield variables when the data for all treatments were pooled, indicating the potential for higher biomass production. However, ‘KX4-Hawaii’ was still productive under the drought conditions seen during the study and future dry conditions may not hamper the generation of useful woody biomass for agronomic purposes. Elfeel & Elmagboul (2016) also found that the rate of Leucaena biomass production was seasonal, similar to the correlation between precipitation and biomass yields in the current work. So, lower green biomass production under drought conditions was expected as reduced leaf production and increased leaf senescence in Leucaena are typical under these conditions (El-Juhany & Aref, 1999; Rosecrance, 1990). However, as woody biomass was more responsive to precipitation than green biomass, which was not significantly correlated with precipitation when the spacing treatments were analysed independently, woody biomass may make up an even larger fraction of the total biomass produced by ‘KX4-Hawaii’ under the climatological norm in Barbados as seen from the January 2019 results.

### Limitations of study and future work

Several constraints were encountered during the study period that were outside the control of the authors. First, Barbados is a small island developing state with limited land availability for agroforestry, and there was limited planting material, so the study was restricted to intra-row plant spacing and the pruning regime was fixed. Second, the study had to be terminated prematurely due to national COVID-19 lockdowns. Hedgerow studies typically are run for at least 2 years and include the effect of pruning management such as that by Mullen et al. (2003). The current study lasted 16 months from the first cut to the last pruning. Nonetheless, the study provides valuable information on ‘KX4-Hawaii’, a new cultivar that has not been significantly studied, on a small island with limited land area and creates a base for future research. Future work will build on this research with experiments of longer durations investigating the effects of pruning management on the cultivar and the application of the biomass produced to the soil.

## Conclusions

Propagation of Leucaena ‘KX4-Hawaii’ *via* rooted air layers was successful and can be recommended as a low-tech method of propagating this seedless cultivar. A plant spacing of 75 cm was superior to that of 50 and 100 cm for promoting ‘KX4-Hawaii’ growth. It resulted in similar biomass yields per meter of hedgerow to a spacing interval of 50 cm, and greater productivity than the 100 cm spacing treatment, but requires less propagation material to establish. ‘KX4-Hawaii’ was still productive when pruned approximately every 4 months under drought conditions. Contrary to some other Leucaena cultivars, woody biomass comprised a larger fraction of the total ‘KX4-Hawaii’ biomass produced with a 4-month pruning interval. Growth and biomass yields were correlated with precipitation, and woody biomass production was more responsive to precipitation. Our current results also suggest that ‘KX4-Hawaii’ is drought tolerant. To the best knowledge of the authors this is the first attempt at understanding the agronomic behavior of ‘KX4-Hawaii’ outside Hawaii and in another island state. Future work is still required to explore the effects of the pruning regime on biomass yields and the ability of ‘KX4-Hawaii’ biomass to promote the growth of a test crop when applied to the soil.

## Supplemental Information

10.7717/peerj.18201/supp-1 Supplemental Information 1 Recorded *Leucaena* growth and biomass data.

## Additional Information and Declarations

### Data Availability

The following information was supplied regarding data availability: The data are available in the Supplemental File.

## References

[ref-1] Ahmad N (2011). Soils of the Caribbean.

[ref-2] Alleyne B (2020). Driest year since 1947.

[ref-3] Bageel A, Honda MDH, Carrillo JT, Borthakur D (2020). Giant leucaena (Leucaena leucocephala subsp. glabrata): a versatile tree-legume for sustainable agroforestry. Agroforestry Systems.

[ref-4] Brewbaker JL (2013). ‘KX4-Hawaii’, seedless interspecific hybrid Leucaena. HortScience.

[ref-5] Brewbaker JL (2016). Breeding Leucaena: tropical multipurpose leguminous tree. Plant Breeding Reviews.

[ref-6] Budelman A (1988). The decomposition of the leaf mulches of Leucaena leucocephala Gliricidia sepium and Flemingia macrophylla under humid tropical conditions. Agroforestry Systems.

[ref-7] Carrington S (2007). Wild plants of Barbados.

[ref-8] Central Bank of Barbados (2023). Mitigating food insecurity in the Caribbean: strategies for a sustainable future. https://www.centralbank.org.bb/news/caribbean-economic-forum/mitigating-food-insecurity-in-the-caribbean-strategies-for-a-sustainable-future.

[ref-9] Chotchutima S, Kangvansaichol K, Tudsri S, Sripichitt P (2013). Effect of spacing on growth, biomass yield and quality of Leucaena (Leucaena leucocephala (Lam.) de Wit.) for renewable energy in Thailand. Journal of Sustainable Bioenergy Systems.

[ref-10] El-Juhany LI, Aref IM (1999). Growth and dry matter partitioning of Leucaena leucocephala (Lam. De Wit.) trees as affected by water stress. Alexandria Journal of Agricultural Research.

[ref-11] Elfeel AA, Elmagboul AH (2016). Effect of planting density on leucaena leucocephala forage and woody stems production under arid dry climate. International Journal of Environmental & Agriculture Research.

[ref-12] FAO (2015). FAO AQUASTAT: country profile—Barbados.

[ref-14] Gallucci M (2019). GAMLj: general analyses for linear models. 1. 2.6.3 ed.

[ref-15] Guevarr AB (1976). Management of Leucaena leucocephala (Lam.) de Wit for maximum yield and nitrogen contribution to intercropped corn. PhD dissertation, The Univeristy of Hawaii, Hawaii, USA. https://scholarspace.manoa.hawaii.edu/items/e07c171a-a3b5-4366-82fe-c85ef8b4bebb.

[ref-16] Idol T, Youkhana A, Santiago RP (2019). Vegetative and micropropagation of Leucaena. Tropical Grasslands-Forrajes Tropicales.

[ref-17] Kang BT, Atta-krah AN, Reynolds L (1999). Alley farming.

[ref-18] Kormawa PM, Kamara AY, Jutzi SC, Sanginga N (1999). Economic evaluation of using mulch from multi-purpose trees in maize-based production systems in South-Western Nigeria. Experimental Agriculture.

[ref-19] Kugedera AT, Mandumbu R, Nyamadzawo G (2022). Rainwater harvesting and Leucaena leucocephala biomass rates effects on soil moisture, water use efficiency and Sorghum bicolor [(L.) Moench] productivity in a semi-arid area in Zimbabwe. Journal of the Science of Food and Agriculture.

[ref-20] Lalljee B (2013). Mulching as a mitigation agricultural technology against land degradation in the wake of climate change. International Soil and Water Conservation Research.

[ref-21] Layne D (2021). Barbados climate data 1991–2020.

[ref-22] Madden M (2023). Fuel bill nearly doubles, food bill also sees major jump.

[ref-23] Maslekar AR (1984). Biomass production in rainfed and irrigated Subabul Leucaena leucocephala plantations. Indian Forester.

[ref-24] Mohan S, Clarke RM, Chadee XT (2020). Variations in extreme temperature and precipitation for a Caribbean island: Barbados (1969–2017). Theoretical and Applied Climatology.

[ref-25] Mullen BF, Gabunada F, Shelton HM, Stür WW (2003). Agronomic evaluation of Leucaena. Part 2. Productivity of the genus for forage production in subtropical Australia and humid-tropical Philippines. Agroforestry Systems.

[ref-26] Noulèkoun F, Khamzina A, Naab JB, Lamers JPA (2017). Biomass allocation in five semi-arid afforestation species is driven mainly by ontogeny rather than resource availability. Annals of Forest Science.

[ref-27] Orwa C, Mutua A, Kindt R, Jamnadass R, Simons A (2009). Agroforestree database: a tree reference and selection guide version 4.0.

[ref-28] Prasad JVNS, Korwar GR, Rao KV, Mandal UK, Rao GR, Srinivas I, Venkateswarlu B, Rao SN, Kulkarni HD (2011). Optimum stand density of Leucaena leucocephala for wood production in Andhra Pradesh, Southern India. Biomass and Bioenergy.

[ref-13] Prosea Foundation (1997). Plant resources of South-East Asia 11, Auxiliary plants.

[ref-29] Proverbs G (1984). Leucaena: ‘A Versatile Plant’.

[ref-30] Rosecrance RC (1990). Leaflet drop in the Leucaena genus. Leucaena Research Reports.

[ref-31] Smith K (2020). Dry spell ‘To Drag On, Effects to Worsen’.

[ref-32] Srivastava R, Singh KP (2013). Implications of multipurpose tree leaf application on wheat productivity in dry tropics. Journal of Forestry Research.

[ref-33] Swinscow TDV (1997). Statistics at square one.

[ref-34] The World Bank (2024). Arable land (hectares)—Barbados. https://data.worldbank.org/indicator/AG.LND.ARBL.HA?locations=BB.

[ref-35] Tian G, Brussaard L, Kang BT (1995). An index for assessing the quality of plant residues and evaluating their effects on soil and crop in the (Sub-) humid tropics. Applied Soil Ecology.

[ref-36] Tuncay T, Rüstü H (1989). The effects of intra-row spacings and cutting heights on the yield of Leucaena leucocephala in Adana, Turkey. Journal of Range Management.

[ref-37] Vargas-Tierras Y, Díaz A, Caicedo C, Macas J, Suárez-Tapia A, Viera W (2021). Benefits of legume species in an agroforestry production system of yellow pitahaya in the Ecuadorian amazon. Sustainability.

[ref-38] Verheij E (2007). Agroforestry.

[ref-39] Youkhana A, Idol T (2008). First-year biomass production and soil improvement in Leucaena and Robinia stands under different pollarding systems. Journal of Tropical Forest Science.

[ref-40] Youkhana A, Idol T (2009). Tree pruning mulch increases soil C and N in a shaded coffee agroecosystem in Hawaii. Soil Biology and Biochemistry.

[ref-41] Youkhana AH, Idol TW (2016). Leucaena-KX2 mulch additions increase growth, yield and soil C and N in a managed full-sun coffee system in Hawaii. Agroforestry Systems.

[ref-42] Youkhana AH, Idol TW, Dagar JC, Tewari VP (2017). Cut-and-carry for sustaining productivity and carbon sequestration in agroforestry systems: coffee-leucaena example. Agroforestry: Anecdotal to Modern Science.

[ref-43] Zanaga D, Van De Kerchove R, Daems D, De Keersmaecker W, Brockmann C, Kirches G, Wevers J, Cartus O, Santoro M, Fritz S, Lesiv M, Herold M, Tsendbazar NE, Xu P, Ramoino F, Arino O (2022). ESA WorldCover 10 m 2021 v200. https://viewer.esa-worldcover.org/worldcover/.

